# Comparative analysis of plant carbohydrate active enZymes and their role in xylogenesis

**DOI:** 10.1186/s12864-015-1571-8

**Published:** 2015-05-22

**Authors:** Desre Pinard, Eshchar Mizrachi, Charles A Hefer, Anna R Kersting, Fourie Joubert, Carl J Douglas, Shawn D Mansfield, Alexander A Myburg

**Affiliations:** Department of Genetics, Forestry and Agricultural Biotechnology Institute (FABI), University of Pretoria, Private bag X20 Hatfield, Pretoria, 0028 South Africa; Centre for Bioinformatics and Computational Biology, Genomics Research Institute (GRI), University of Pretoria, Private bag X20 Hatfield, Pretoria, 0028 South Africa; Evolutionary Bioinformatics Group, Institute for Evolution and Biodiversity, Hufferstr. 1, Munster, D48149 Germany; Department of Botany, University of British Columbia, 6270 University Blvd, Vancouver, BC V6T 1Z4 Canada; Department of Wood Science, University of British Columbia, 2424 Main Mall, Vancouver, BC V6T 1Z4 Canada

**Keywords:** Carbohydrate active enzymes, Comparative genomics, Transcriptomics, *Eucalyptus grandis*, Protein domains, Wood formation, *Populus trichocarpa*

## Abstract

**Background:**

Carbohydrate metabolism is a key feature of vascular plant architecture, and is of particular importance in large woody species, where lignocellulosic biomass is responsible for bearing the bulk of the stem and crown. Since Carbohydrate Active enZymes (CAZymes) in plants are responsible for the synthesis, modification and degradation of carbohydrate biopolymers, the differences in gene copy number and regulation between woody and herbaceous species have been highlighted previously. There are still many unanswered questions about the role of CAZymes in land plant evolution and the formation of wood, a strong carbohydrate sink.

**Results:**

Here, twenty-two publically available plant genomes were used to characterize the frequency, diversity and complexity of CAZymes in plants. We find that a conserved suite of CAZymes is a feature of land plant evolution, with similar diversity and complexity regardless of growth habit and form. In addition, we compared the diversity and levels of CAZyme gene expression during wood formation in trees using mRNA-seq data from two distantly related angiosperm tree species *Eucalyptus grandis* and *Populus trichocarpa*, highlighting the major CAZyme classes involved in xylogenesis and lignocellulosic biomass production.

**Conclusions:**

CAZyme domain ratio across embryophytes is maintained, and the diversity of CAZyme domains is similar in all land plants, regardless of woody habit. The stoichiometric conservation of gene expression in woody and non-woody tissues of *Eucalyptus* and *Populus* are indicative of gene balance preservation.

**Electronic supplementary material:**

The online version of this article (doi:10.1186/s12864-015-1571-8) contains supplementary material, which is available to authorized users.

## Background

Carbohydrate metabolism in plants is responsible for a diverse array of developmental processes, including energy metabolism, signaling, defense, cell wall (CW) structure [1], and carbohydrate-related post-translational modifications [2]. Carbohydrate biopolymers in the secondary cell walls (SCWs) of fiber cells form the bulk of woody biomass, a valuable natural resource with a variety of industrial applications, including pulp and paper, and potential biofuel production [3,4]. The vessel and fiber cells in angiosperm wood have significant amounts of cellulose, hemicellulose and lignin in their SCWs [5,6]. Cellulose and hemicelluloses are synthesized, modified, and degraded by Carbohydrate-Active enZymes (CAZymes), a group comprising of modular protein domains that are ubiquitous across all living organisms [7-9]. CAZymes have been classified into four classes of enzymatic domains, namely glycosyl transferases (GTs), glycoside hydrolases (GHs), polysaccharide lyases (PLs) and carbohydrate esterases (CEs) [9,10]. In addition, a non-enzymatic class exists, the carbohydrate-binding modules (CBMs) [11,12]. Currently, the five CAZyme classes are collected and organized into families based on amino acid sequence similarity in the CAZy database [8,13].

GTs catalyze glycosyl bonds between a donor sugar substrate and another molecule, typically another sugar [14]. Along with defense, signaling and storage carbohydrate biosynthesis, plant GTs are responsible for the production of cellulose (GT2 domain family- *Cellulose synthase A* (*CESA*) gene superfamily) [15] and hemicelluloses (GT2, GT8, 43, 47, and 61 families, among others) [16-21]. GH domains hydrolyze the glycosyl bonds between sugars in carbohydrate biopolymers [9]. They play an important role in the modification of biopolymers to be introduced into the CW, as well as abscission and dehiscence [22]. PLs are implicated in non-hydrolytic cleavage of activated glycosidic bonds in pectin modification and degradation [23,24]. CEs de-acetylate polysaccharide side-chains, and are thought to modify the cross-linking of hemicellulose with lignin [25,26]. CBMs allow for specific binding to different carbohydrate biopolymers, facilitating precise biopolymer modifications by enzymatic domains as they are added to the CW [27,28]. Due to their ability to disrupt the SCW network by binding to CW polymers, CBMs have been used in industry to increase the efficiency of CW degradation during the pulping process [29].

Previous studies have found higher frequency and diversity of CAZyme genes in the genome of *P. trichocarpa* than that of *A. thaliana* [30], which in 2001, had the most annotated CAZymes in its genome compared to sequenced fungal and bacterial species [11]. Furthermore, the CAZymes expressed in wood-forming tissues of *P. trichocarpa*, specifically those involved in cellulose and hemicellulose biosynthesis, are more abundant and diverse than those in non-wood forming tissue such as young leaves [30]. Based on these findings, the authors noted the importance of CAZymes to woody characteristics.

Protein domains, as the functional and evolutionary building blocks of plant proteins, are informative of the functional capacity of the genome [31,32]. A recently published database of CAZyme domains, dbCAN [33,34], can be employed to identify the frequency and diversity of CAZyme domains in plant genomes available on Phytozome [35]. dbCAN utilizes Hidden Markov Models (HMMs), based on the seminal CAZyme family sequence data available [13], to accurately and reproducibly identify CAZyme domains [33]. Using the dbCAN database, protein-coding genes containing CAZyme domains in twenty-two plant species can be compared to analyze their CAZyme domain repertoire.

Fundamental questions about the evolution of woody plant secondary growth still exist [36], and with the availability of the genome of a second hardwood species, that of *E. grandis,* along with mRNAseq data for *E. grandis* and *P. trichocarpa* [37], some of these questions can be addressed. We aimed to characterize the CAZyme domain frequency and diversity in plant species, and their expression levels in *P. trichocarpa* and *E. grandis* woody and non-woody tissues. In this way, we could identify the common expressed CAZyme repertoires involved in carbohydrate metabolism in wood-forming tissues of two evolutionary divergent tree genera. Specifically, we asked: Does the frequency and diversity of CAZyme domains between plants reflect their evolution and developmental complexity? Is the expression of CAZyme domains related to wood formation in *E. grandis* and *P. trichocarpa*? We hypothesized that the genomes of woody trees would exhibit gains or expansions of CAZyme domain containing genes that contribute to carbohydrate biopolymer formation and deposition in wood formation. Further hypotheses were that the expression investment of CAZyme domain-containing genes abundant in the developing xylem would be higher than in non-xylogenic tissue as a reflection of focused carbohydrate metabolism in this sink tissue. This study is the first systematic analysis of genome-wide and expressed CAZyme domains across a diversity of plant species, with a focus on CAZyme utilization for the production of lignocellulosic biomass.

## Results

### Genome-wide analysis of CAZyme classes in plants

**Figure 1:**
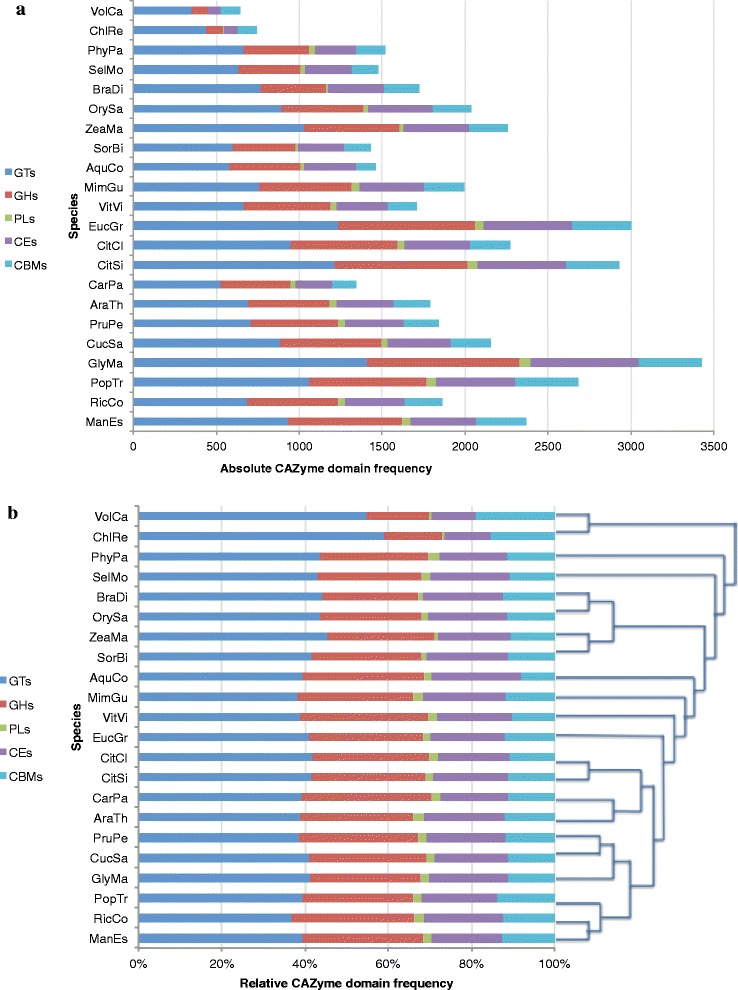
Absolute and relative frequency of CAZyme domain class frequency across twenty-two plant species. **(a)** Absolute frequency of CAZyme domains in five classes across twenty-two plant species. Plant species are on the y-axis, and the absolute frequency of CAZyme domains within all CAZyme genes is shown on the x- axis. The glycosyl transferase (GT) domain class is represented in blue, glycosyl hydrolase (GH) domain class in red, polysaccharide lyase (PL) domain class in green, carbohydrate esterase (CE) domain class in purple and carbohydrate binding module (CBM) domain class in light blue. **(b)** Relative frequency of CAZyme domain classes in twenty-two plant species. The relative frequency of carbohydrate active enzyme (CAZyme) domain classes in CAZyme genes, as a percentage, is shown on the x-axis. The species of plant is shown on the y-axis. For species abbreviation, refer to Table 1.

To gain insight into the evolution of CAZyme genes across key land plant evolutionary lineages, we compared the domain content of twenty-two plant species that have been annotated in dbCAN from Phytozome by examining the number of genes containing CAZyme domains, and the frequency of CAZyme domains in these genes within each plant genome (Table 1). The absolute frequency of genes from each CAZyme class per genome shows that seed producing plants (except *Carica papaya*- 1,341 CAZyme domains) have more CAZyme containing genes and CAZyme domains than non-seed plants such as the bryophyte *Physcomitrella patens* (1,519 CAZyme domains) and the lycophyte *Selaginella moellendorffii* (1,476 CAZyme domains), and almost double that of the green algal species *Volvox carteri* and *Chlamydomonas reinhardtii* (654 and 731 CAZyme domains, respectively) (Figure 1a, Table 1). However, the absolute frequencies of these genes in angiosperms can be misleading, as some plant genomes have undergone one or more whole genome duplications (WGD) and experienced extensive gene loss in the past [38-40]. The absolute gene frequency may reflect the age of the genome since the last WGD and the rate of gene loss in the lineage, as well as other mechanisms such as gene retention after neo/sub-functionalization, or tandem gene duplication [38,39,41].

**Table 1:** **Genome- wide CAZyme gene and domain content for twenty-two plant species**

Organism*	**Genome Size (Mbp)**	**#Genes**	**#CAZyme genes**	**%CAZyme genes**	**#CAZyme domains**	**# GTs**	**# GHs**	**# PLs**	**# CEs**	**# CBMs**	**Reference**
***Volvox carteri*** **(VolCa)**	138	14,520	490	3.37	645	283	85	4	65	102	[67]
***Chlamydomonas reinhardtii*** **(ChlRe)**	121	15,143	574	3.79	741	367	85	4	76	87	[68]
***Physcomitrella patens*** **(PhyPa)**	480	35,938	1,236	3.44	1,519	664	392	41	250	172	[69]
***Selaginella moellendorffii*** **(SelMo)**	212	22,285	1,224	5.49	1,476	637	370	27	281	161	[70]
***Brachypodium distachyon*** **(BraDi)**	272	25,532	1,418	5.55	1,723	764	394	20	334	211	[71]
***Oryza sativa*** **(OrySa)**	420	42,109	1,724	4.09	2,040	891	498	29	389	233	[72]
***Zea mays*** **(ZeaMa)**	2,300	30,579	1,920	6.28	2,256	1,026	578	22	394	236	[73]
***Sorghum bicolor*** **(SorBi)**	730	34,496	1,751	5.08	1,431	784	474	23	288	171	[74]
***Aquilegia coerulea*** **(AquCo)**	302	24,823	1,554	6.26	1,464	657	471	32	360	141	[75]
***Mimulus guttatus*** **(MimGu)**	312	26,718	1,671	6.25	1,992	680	503	43	363	201	[76]
***Vitis vinifera*** **(VitVi)**	490	30,434	1,424	4.68	1,710	664	525	39	305	177	[77]
***Eucalyptus grandis*** **(EucGr)**	641	36,376	2,542	6.99	3,334	1,233	823	54	534	360	[37]
***Citrus clementina*** **(CitCl)**	301	24,533	1,971	8.03	2,328	862	572	41	413	205	[78]
***Citrus sinensis*** **(CitSi)**	319	25,376	2,439	9.61	2,927	1,049	735	52	498	267	[78]
***Carica papaya*** **(CarPa)**	372	27,873	1,131	4.06	1,341	466	380	25	209	124	[79]
***Arabidopsis thaliana*** **(AraTh)**	119	27,400	1,505	5.49	1,787	697	483	49	341	217	[80]
***Prunus persica*** **(PruPe)**	227	27,852	1,591	5.71	1,843	654	491	34	337	180	[81]
***Cucumis sativus*** **(CucSa)**	243	26,682	2,157	8.08	2,157	779	555	36	355	184	[82]
***Glycine max*** **(GlyMa)**	975	54,175	2,839	5.24	3,429	1,412	917	69	645	386	[83]
***Populus trichocarpa*** **(PopTr)**	422	41,335	2,252	5.45	2,677	1,057	713	55	479	373	[84]
***Ricinus communis*** **(RicCo)**	350	31,237	1,540	4.93	1,864	605	486	36	328	186	[85]
***Manihot esculenta*** **(ManEs)**	533	30,666	1,957	6.38	2,365	825	616	42	377	239	[86]

*The first column shows the plants analysed with the abbreviations used in this study.

Although the absolute frequencies of CAZy domains vary between plant genomes, we found that the proportions of the five functional CAZymes classes (GT, GH, CE, PL, CBM) are remarkably similar among species (Figure 1 a, b). A co-efficient of variance analysis was performed to determine if the ratios of CAZyme classes among monocots, eudicots, lycophytes and bryophytes, and green algae varied significantly. For all CAZyme domain classes except PLs, the difference in the frequency ratios among the land plant (excluding the green algae) classes was negligible (Additional file 1: Table S1). In land plants, GTs comprised roughly 40% of the CAZyme domain content in the genome, with GHs having a relative frequency of 30%. CEs, PLs and CBMs have relative frequencies of 18, 2 and 10 percent, respectively. In contrast, the green algae have frequency ratios of approximately 57% for GTs, 14.5% for GHs, 0.6% for PLs, 10% for CEs and 17% for CBMs.

### Genome-wide comparison of CAZy domain diversity and complexity

Next, we asked what the diversity of CAZyme families within each class is between plant species. Here the objectives were to analyze the CAZyme domain families within each broader functional class present in each genome to determine whether the presence of unique domain families contributed to the organismal complexity of vascular and/or seed producing plants. All twenty-two species from the previous analysis were analyzed to determine the diversity of the domain families present in each species (Additional file 2, Table 1). There are 231 different CAZyme domains present across the plant lineages analyzed (72 GT, 92 GH, 13 PL, 16 CE and 38 CBM families). The frequency of all families across the 22 species displayed a bimodal distribution (R^2^ = 0.68), either present in all species, ubiquitous to all land plants considered, or present only in one or two species (Additional file 3: Figure S1). This bimodal pattern has been observed more generally in the context of shared domain combinations among plants, and consistent with large shared domain gain events early in land plant evolution [32]. There are 74 domain families that are found in one or two species are representatives from the GT, GH, PL and CBM classes, with none from the CE class (Additional file 3: Figure S1, Additional file 2). 5 CBMs, 1 GH and 5 GTs are found exclusively in the green algae, while the lycophytes and bryophytes share one GH that only occurs in those species. The rest are interspersed amongst the remaining 18 vascular seed plants. CBM17 is only found in the two citrus species, and is thought to bind to amorphous cellulose (http://www.cazy.org/CBM17.html).

Of the 231 CAZyme domain families found across all 22 species, only 65 are common to all, and 20 occur ubiquitously across all land plants, but are absent in the green algae (Additional file 2). However, within all genomes, the frequency of these 65 conserved families account for the majority (75% ± 2%) of CAZyme domains, with the 20 land plant-specific domains accounting for an additional 12%-17% of CAZyme domains in these species. Only one CAZYme domain, CBM16, was observed to occur ubiquitously across seed plants, but absent in the lycophyte *Selaginella moellendorffii* and moss *Physcomitrella patens,* and the green algae *Chlamydomonas rienhardtii* and *Volvox carteri* (Additional file 2). CBM16, which is known to bind to cellulose and glucomannan, is only detected in the eighteen seed plants in the twenty-two species analyzed, although it should be noted that it is also found in Archaea and Bacteria [13]. In *Eucalyptus* and most other seed plant species, this CBM occurs exclusively in DUF642-domain containing proteins. The biological function of these proteins has not yet been fully resolved but they have been demonstrated to be essential for various aspects of developmental biology (see [42] for a recent review). The domain has been previously reported to be seed-plant specific, but a search revealed it is also present in conifers [43,44]. There were no unique domains in *A. thaliana*, and there were no domains that were unique to the two woody perennials, *E. grandis* and *P. trichocarpa* compared to the other plant species analyzed.

The distribution of CAZy domain-containing multi-domain proteins in ten representative land plant species (eight seed plants, *S. moellendorffii* and *P. patens*) followed the power law of gene complexity and gene number ([45], Additional file 4: Figure S2). The composition of complex CAZyme proteins was considered in terms of whether they consisted solely of repeat domains, or of combinations of different domains. An average of 17% of CAZyme proteins were found to contain repeat domains in all 10 genomes considered, of which 60% are repeats of the same CAZyme domain (Additional file 5). All CAZy domain-containing proteins that have five or more domains in all species examined contain GT41 (O-linked β-N-acetylglucosamine transferase) domains, which are involved in post-translational (O-GlcNAc) protein modification [46,47].

Proteins consisting of more than one CAZyme domain show lineage specific combinations across the ten plant genomes analyzed. In the five eudicots considered, 15 CAZyme domain combinations are common to all five (Additional file 6: Figure S3). *Glycine max* has the most unique combinations between the eudicots at six, with *P. trichocarpa*, *E. grandis* and *V. vinifera* having two and *A. thaliana* having only one. Of the 28 CAZyme domain combinations that occur in *E. grandis*, the six that have genomic frequency greater than 10 are: CBM43-GH17, GH28-GH55, CBM18-GH19, CBM22-GH10, CE1-CE10 and CE1-CE7 (Additional file 7). Similarly in *P. trichocarpa*, of the 25 CAZyme domain combinations that occur more than 10 times in the genome are CBM43-GH17, CBM18-GH19 and GH28-GH55 (Additional file 7). CBMs in combination are thought to act as enhancers and mediators of the enzymatic action of their appended domains. In the *E. grandis* dataset, this co-operative relationship is evident in the activity of the enzymatic domain and the specificity of the attached CBM. For example, CBM 43 binds to β-1,3-glucan, and GH17 is a β-1,3-endoglucanase [13]. Similarly, CBM22 binds specifically to xylan and GH10 is a xylanase. Combinations with CBM domains are prevalent in the *E. grandis* genome, with CBM to enzymatic CAZyme domain combinations accounting for 11 of the 28 combinations.

### Expression of CAZyme domain containing genes in *E. grandis*

**Figure 2:**
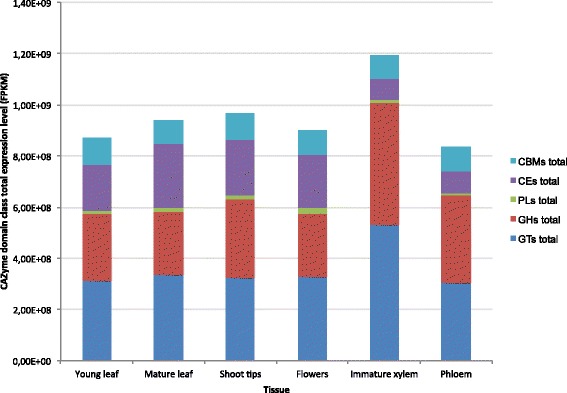
Total gene expression levels of five CAZyme domain classes across six tissues in *E. grandis*. The y-axis represents the mRNA-Seq expression data in FPKM, and the x-axis the tissue type analyzed. The average expression data in FPKM for each gene can be found in Additional file 2.

The newly sequenced genome of *E. grandis* [37] as well as RNA-sequencing data for several tissues and organs [48] allowed a functional genomics investigation of CAZyme containing genes. Expression profiling across six tissues in *E. grandis* showed that of the 2,542 CAZyme-domain containing genes in the *E. grandis* genome, 80.5% (2,044) are expressed in at least one tissue (Additional file 8). The relative proportions of transcript abundance for each CAZyme domain class were similar across tissues (Figure 2), although the expression of GH and GT domain classes were proportionally higher in the immature xylem. GTs constitute 44.5% of expression investment of CAZyme domain containing genes in the immature xylem *vs.* 35.9% in the young leaf. GHs account for 39.8% of the transcript abundance of CAZyme domain containing genes in the immature xylem, and 29.7% in the young leaf (Figure 2). CE domain family expression was proportionally lower in the phloem and immature xylem compared to the young leaf, mature leaf, flowers and shoot tips, making up 7% of the total CAZyme expression investment in the immature xylem and 20.3% in the young leaf. Variation at the level of individual CAZyme domain families was observed, and is discussed below.

**Figure 3:**
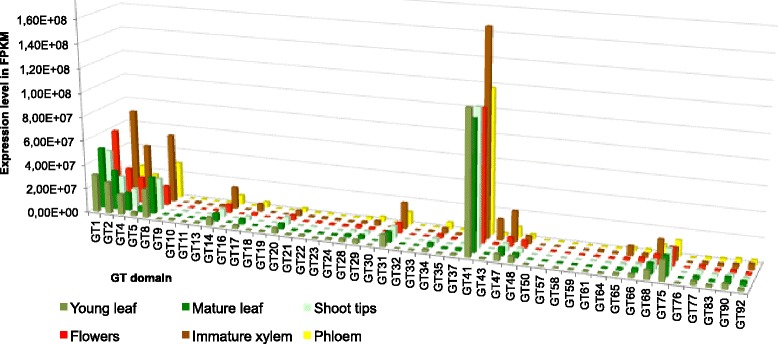
Total gene expression levels of GT domain families across six tissue types in *E. grandis*. The y-axis shows the total expression investment in FPKM from raw mRNA-Seq data summed across expressed glycosyl transferase (GT) genes, while the x-axis shows the GT domain family. The depth axis is the tissue type in *E. grandis* for which each domain family expression level in FPKM was calculated. Light green- young leaf, dark green- mature leaf, mint green- shoot tips, red- flowers, brown-immature xylem and yellow- phloem. The expression level in FPKM for each gene can be found in Additional file 8.

The majority of GT domain families showed fairly low levels of ubiquitous expression across six tissues in *E. grandis* tissues (Figure 3, Additional file 9). We identified domain families that had expression levels higher in a single tissue compared to the remaining five. Of the forty-seven GT domain families present in the *E. grandis* genome, nine GT families (GT1, GT2, GT4, GT8, GT14, GT31, GT41, GT43, and GT47)(Figure 3) accounted for 80% of GT expression in immature xylem (Additional file 9). GT41 had the highest expression investment across all tissues (Figure 3). GT41 proteins often contain repeats of the GT41 domain, and in *E. grandis* the gene (Eucgr.L00641) contains seven GT41 repeats and had the highest expression of all GT41 containing proteins at >6 million FPKM in the xylem (Additional file 8). The GT41 domain occurs 241 times in the *E. grandis* genome, of which 120 genes containing this domain are expressed in at least one tissue. In comparison, GT1 occurs 511 times in the genome, of which 332 were expressed in at least one tissue and had lower expression investment in the immature xylem compared to the other five tissues analyzed. Thus, GT1 domains are more prevalent in the genome, and more genes containing this domain were expressed, but the magnitude of expression of these genes was considerably lower than the less abundant GT41 domain containing genes.

GH domain family expression investment across six tissues in *E. grandis* (Additional file 10: Figure S4, Additional file 9) showed that three GH domain families (GH9, GH16 and GH19) showed relatively high levels of expression in different tissues. GH16 domain containing genes were highly expressed in the immature xylem and phloem, and GH19 domain containing genes were highly expressed in the immature xylem and shoot tips. The GH16 domain family is present in the xyloglucan endotransglycosylase/transferase (XTH) gene family, which contribute to side chain hydrolysis or side chain re-arrangement without hydrolysis (Eklöf *et al.*, 2013). GH9 domain families were also preferentially expressed in the immature xylem compared to the other tissues, the overall higher expression investment in the immature xylem being due to fewer genes (18) being expressed at higher levels than in the other tissues, similar to the GT41 domain containing genes (Additional file 10: Figure S4, Additional file 9). The most highly expressed CAZyme gene in *E. grandis* xylem was a GH19 family gene, *Eucgr.H04034* at 1,01E + 08 FPKM (Additional file 8). The *A. thaliana* ortholog *AT3G16920* is a chitinase-like (*CTL2*) gene, which is known to be involved in cellulose synthesis [49].

PL families have relatively few CAZyme domain families (13) across all species, including the four expressed in *E. grandis*. The expression investment of PL domain families across six tissues in *E. grandis* showed that all four PL domain families are expressed at diverse levels in all tissues (Additional file 11: Figure S5). PL1 and PL10 showed high expression investment in the flowers compared to the other five tissues. There were no PL families that showed high expression in woody tissues compared to non-woody tissues. CEs showed interesting expression investment across six tissues in *E. grandis* in that they were expressed fairly ubiquitously at the same level across all tissues (Additional file 12: Figure S6), leading to their lower proportional expression investment level in the immature xylem compared to GT and GH expression investment. Of the 12 CE domain families that were expressed in the *E. grandis* genome, most were expressed at the same level across the different tissues. The exception to this pattern is CE16, which had low relative expression in the immature xylem and phloem, despite having the highest level of expression across the remaining four tissues of all the CE families. CE16 domain-containing genes are acetyl xylan esterases which de-acetylate preferentially at the *O-3* and *O-4* positions of the backbone xylopyranosyl residues [50].

Most CBM domain families did not show preferential expression investment, and were expressed at the same (relative) level in three or more tissues (Additional file 13: Figure S7). There are two exceptions: CBM18 (chitin-binding), which is highly expressed in young leaf and shoot tips compared to the other four tissues, and CBM22 (xylan binding), which is highly expressed in the immature xylem compared to the other five tissues. CBM57, which is also the most abundant CBM in both *E. grandis* and *P. trichocarpa*, showed the highest expression investment of all the CBM domains expressed in the mature leaf, immature xylem and the shoot tips, while CBM43 has the highest expression investment in the flowers and phloem. CBM43 and CBM57 together contributed 51% of the total average expression investment out of 17 CBM domain families in all tissues. CBM57 was first described in malectin [51], and is involved in the recognition of Glc2-N-glycans. In *E. grandis*, all of the proteins containing these domains are kinases, including mainly LRR protein kinases. Some of the ones highly expressed in wood include HERK1 and THESEUS (brassinosteroids responsive and required for cell elongation during vegetative growth) [52,53].

### Comparative expression investment of CAZyme domains in *E. grandis* and *P. trichocarpa*

To assess the expression investment of CAZyme domain families in two distantly related angiosperm tree species, we compared the transcript abundance of CAZyme domain families in *E. grandis* and *P. trichocarpa* xylem and leaf tissue (Additional file 9). The pattern of CAZyme family expression was considered to be the same if the family was expressed higher in the xylem than in the leaf in both species and vice versa. The absolute transcript abundance in FPKM cannot be directly compared between these analyses as the experiments were conducted independently, with gene length (K) and sequence depth (M) parameters normalized within the individual transcriptomes for each species. Relative expression values in each tissue can however be compared to identify common expression patterns between the two species.

**Figure 4:**
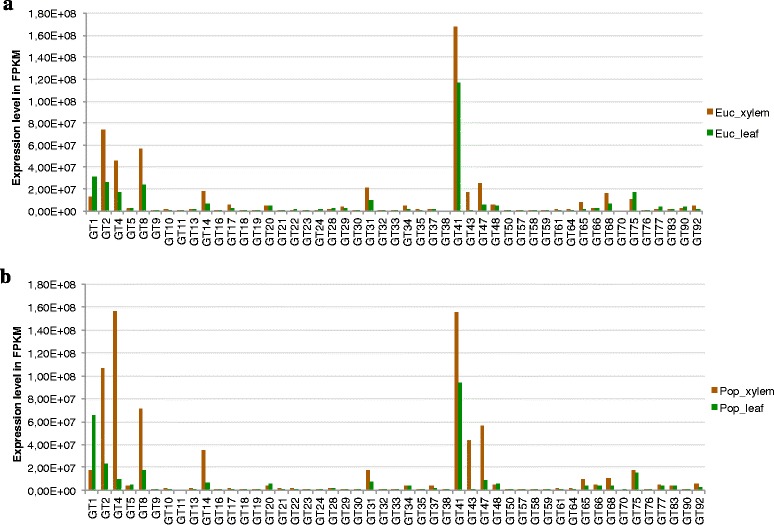
Total gene expression levels of GT domain families in *E. grandis* and *P. trichocarpa* xylem and leaf tissues. **(a)** Expression level per glycosyl transferase (GT) family in *E. grandis* in xylem and leaf tissues. The y-axis shows the transcript abundance in FPKM, the x-axis shows the GT family with xylem in brown, and leaf in green. **(b)** GT domain family (x-axis) expression level for *P. trichocarpa* xylem and leaf tissues in FPKM (y-axis).

For the GT family of CAZyme genes, the expression pattern was similar in *E. grandis* and *P. trichocarpa*. The majority of GT domain families were expressed at a low level in *E. grandis* and *P. trichocarpa* xylem and leaf tissue, which indicates that they are involved in other aspects of carbohydrate metabolism, rather than CW biosynthesis (Figure 4). GT1 family showed higher expression investment in the leaf tissue as opposed to the xylem tissue in both *E. grandis* and *P. trichocarpa*. The GT domain families identified in this study as contributing to the majority (80%) of expression investment in the immature xylem compared to the other five tissues in *E. grandis* (Figure 3, Additional file 9) showed greater expression investment in both *E. grandis* and *P. trichocarpa* xylem compared to leaf (Figure 4). These include the domain families that have been implicated in cellulose and hemicellulose biosynthesis, namely GT2, GT4, GT8, GT14, GT31, GT43, and GT47. The conservation of these expression investment patterns between source (mature leaves) and sink (immature xylem) tissues of divergent tree species indicates a conserved mechanism for CW biosynthesis at the functional domain level, and highlights the importance of regulation of these genes at the level of transcript abundance.

The expression pattern of GH domains in *E. grandis* and *P. trichocarpa* was similar for most of the GH domain families (Additional file 14: Figure S8a, b). GH4 family was not expressed in the *E. grandis* tissues studied, including the xylem, while it was expressed at relatively low levels in the xylem and leaf tissue of *P. trichocarpa*. Furthermore, GH57, 62 and 80 were not expressed in *P. trichocarpa* xylem and leaf, while they were expressed at low levels in *E. grandis.* GHs that were expressed at low levels in one species and not in the other may be involved in specific defense or response to abiotic factors, and were thus not captured in a tissue transcriptome of either species. As with *E. grandis*, the most highly expressed CAZyme in the immature xylem of *P. trichocarpa* was a CTL2 (GH19 containing) homolog, Potri.010G141600 (Additional file 8).

Comparison of PL family expression between *E. grandis* and *P. trichocarpa* xylem and leaf tissue (Additional file 15: Figure S9a, b), showed that PL1 had a higher expression investment in the xylem as compared to the leaf in both species, with xylem:leaf ratios of 1.9 and 7.1 in *E. grandis* and *P. trichocarpa*, respectively. The same four PL domain families are present in the genome and expressed in both *E. grandis* and *P. trichocarpa*. The CE domain class showed variable expression investment patterns between xylem and leaf tissues for *E. grandis* and *P. trichocarpa* (Additional file 16: Figure S10a, b). CE2, 3 and 5 were not expressed in *E. grandis* and were expressed at relatively low levels in *P. trichocarpa* xylem and leaf. CE15 was expressed in *E. grandis* and not in *P. trichocarpa.* The CE8 domain family contains pectin methylesterase genes (Jolie *et al.*, 2010), and was more highly expressed in *P. trichocarpa* xylem than in the leaf, while *E. grandis* showed the opposite trend (Additional file 16: Figure S10a, b). CE16 had the highest expression investment in the leaf tissue of all the CE domain families expressed in both species. CBMs showed different expression investment patterns between *P. trichocarpa* and *E. grandis* xylem and leaf tissues in a number of families (Additional file 17: Figure S11a, b). Of all the CBMs expressed in *E. grandis* xylem and leaf, CBM18 showed the highest expression investment in the leaf, while CBM57 had the highest expression level in xylem. In *P. trichocarpa*, of all the CBM domain families expressed, CBM47 had the highest expression in both xylem and leaf (Additional file 17: Figure S11a, b).

## Discussion

Our analysis revealed that the genomic content of CAZyme domains in evolutionary diverse plant genomes is conserved with respect to the ratios of CAZyme classes, although the absolute frequencies vary (Figure 1). Our hypothesis, based on previous findings [30], was that woody perennials would possess a larger proportion of GTs for aspects of carbohydrate metabolism needed to support a large commitment to secondary wall biosynthesis during extensive secondary xylem development (wood production). We found that, in fact, all land plants analyzed show a genomic ratio of 4:3 of GTs to GHs, regardless of their relative investment in different types of carbohydrate metabolism. When considering the maintenance of the ratios of the different CAZyme classes within the genomes between plant species, we observe that the ratio of functional enzymatic domains is maintained, despite differences in whole-genome-, tandem- and segmental duplication events between the species considered. Thus, CAZymes and carbohydrate metabolism may be subject to gene dosage balance selection [54,55]. This is also supported by the conservation of relative expression ratios of the majority of CAZyme classes operating in source and sink tissues of *Eucalyptus* and *Populus*.

These results, combined with our examination of the presence of unique domains in the genomes of plants, leads us to conclude that the genomic potential to metabolize carbohydrates for wood production is not associated with the emergence of unique CAZyme domain famies. The fact that *P. patens*, despite relatively low numbers of CAZyme genes, has a larger genomic diversity of CAZyme domain families than almost all vascular plant species considered, indicates that primary cell wall (PCW) and SCW metabolism employs a standard set of CAZyme domains in different tissue types of different land plant species. Within woody species, the diversity of CAZyme domain families may contribute to wood formation via unique combinations and regulatory mechanisms of ancestral domains within the genomic and transcriptomic context. We have found that combinations of CAZyme domains do not differentiate woody plants from non-woody plants, as the majority of the types of domain combinations in complex proteins are common between lineages, with low promiscuity of domains (Additional file 7).

Using a comparative transcriptomics approach we were able to define a global view of carbohydrate metabolism in carbon source and sink tissues, and the conservation of transcriptional regulation of CAZyme domains between the divergent woody trees *Eucalyptus* and *Populus*. The most highly expressed genes in both *E. grandis* immature xylem and *P. trichocarpa* xylem were the GH19 domain-containing genes Eucgr.H04034 and Potri.010G141600, respectively. *CTL2*; along with its homolog *CTL1,* modifies cellulose microfibrils as they are extruded from the CesA complex, as illustrated by the reduced levels of crystalline cellulose in double knockdown mutants of *ctl1/ctl2* [49]. This, as well as the high and preferential expression of GH16 and GH9 containing proteins (e.g. homologs of KOR1 and GH9B7 in both species), highlights the importance of domains responsible for degradation acting as modifiers to the synthesis of SCW cellulose.

GTs known to synthesize cellulose and hemicellulose showed greater expression investment in the immature xylem compared to the other tissues in *E. grandis* (Figure 3). Furthermore, these GT domain families displayed conserved expression patterns in *E. grandis* and *P. trichocarpa* xylem and leaf (Figure 4), indicating the importance of relative stoichiometric conservation of functional carbohydrate enzymatic processes at the transcript level. This pattern of conserved domain expression investment in xylem is seen in GT2, GT4, GT8, GT14, GT31, GT41, GT43, and GT47domain families. GT41 family genes are GlcNAc transferases, involved in a multitude of functions, predominantly intracellular signaling [47]. Signaling provides the sensitive feedback necessary to coordinate the deposition of CW polysaccharides. GT41-mediated modification of proteins can be compared to phosphorylation, as it is a dynamic method of post-translational modification for cytoplasmic and nuclear proteins. GT41 domain containing proteins are also the most complex CAZymes, with greater than four GT41 domain repeats within a single gene found across all plant species analyzed.

GT43 family members *IRX9* and *IRX14*) [56] and GT47 family members (e.g. *FRAGILE FIBER8*) [57] are known to be involved in xylan biosynthesis. GT43 gene family members are responsible for xylan backbone biosynthesis and have conserved biochemical functions across vascular plants [56]. GT8 domain family containing genes showed high expression investment in the immature xylem compared to the other tissues analyzed, and members of this gene family have been characterized as xylan glucuronyl transferases, including the *PARVUS*, *GALACTURONOSYLTRANSFERASE,* and *GALACTURONOSYLTRANSFERASE-LIKE* (*GAUT*/*GATLI*) genes [58,59]. The GT31 domain containing gene *At4g21060* in *A. thaliana* has been shown to encode a galactosyltransferase that is responsible for arabinogalactan protein galactosylation during backbone formation [60]. Amongst the other GTs that displayed preferential expression in immature xylem than the other tissues, we found GT65 and GT68, which are fucosyl and oligosaccharide transferases respectively [13]. GT and GT-like enzymes accounted for 20% of the proteome of *A. thaliana* Golgi apparatus, including GT14, GT8 and GT31 domain containing proteins [61], lending some confidence to the expression abundance observed in this study.

## Conclusions

A key finding of this study is that the CAZyme containing genes in plant genomes have a conserved ratio between species, regardless of their carbohydrate metabolic strategy or the tandem, segmental or documented whole genome duplication events in their evolutionary history. Although we find evidence for lineage specific diversity of CAZyme families in plant genomes, the domain family diversity of CAZymes cannot be used to discriminate the eudicot and monocot lineages, or woody and herbaceous species. The expression pattern of the CAZyme domains responsible for cellulose and xylan biosynthesis appears to be stoichiometrically conserved between *Eucalyptus* and *Populus*. This study highlights the importance of transcriptional regulation in the evolution of wood development as opposed to genomic innovations in the enzymatic domains responsible for carbohydrate metabolism.

## Methods

### Genome-wide analysis of CAZyme domains in plant species

All CAZyme domains for twenty-two plant species (Table 1) in Phytozome v8.0 [35] were obtained from the dbCAN database [33,34]. The plant species examined for the genome-wide analysis of CAZyme domains were chosen in order to encompass the Viridiplantae lineage (see Table 1 for all species and abbreviations), including Chlorophyta (including only *C. reinhardtii* and *V. carteri*), Embryophyta, encompassing *P. patens* onwards, Tracheophyta, encompassing *S. moellendorffii* onwards and monocot and dicot representatives of the Magniliophyta.

Analysis was performed using custom Python scripts (Python v2.6, Additional files 18 and 19). The Python language [62] was used in this study to write and execute custom scripts to rapidly, reproducibly and accurately analyze large tables of data. The primary applications of these scripts included basic data manipulation of the text files obtained from the dbCAN database, firstly extracting all the CAZyme domains present in each genome and collating them by domain family (Additional file 18), and secondly, counting all the CAZyme domains in each family per genome (Additional file 19). These collated and counted values of domain frequency per CAZyme domain family per genome were analyzed further in Excel. We classified three parameters, namely i. Frequency- the absolute numbers and relative frequencies of annotated genes within each of the five CAZyme domain classes, and the families assigned to these classes in the genomes of all twenty-two species, ii. Diversity- the number and type of individual CAZyme domain families within and between species, and iii. Complexity- occurrence, frequency and diversity of CAZyme domains *within* annotated genes. Covariance analysis to determine within and between species CAZyme domain class relative frequency variation was done using SAS v9.3 (Statistical Analysis Software- SAS Institute Inc.).

Diversity of CAZyme domains were analyzed by grouping and counting all the individual domains present in each genome (including *each* domain in multi-domain proteins) into their families based on dbCAN annotations. Complexity analysis was performed on a subset of ten species representing major lineages of land plant evolution (Table 1). The analysis of CAZyme domain complexity with annotated genes in each genome involved identifying all annotated genes that contained multiple CAZyme domains. Firstly, the number of annotated domains per gene in each of the ten genomes was calculated and visualized in Excel. Secondly, all genes containing multiple annotated CAZyme domains were separated based on whether they consisted solely of repeat CAZyme domains, or contained unique CAZyme domain families. These two categories of multiple CAZyme domain containing annotated genes were then analyzed either by the frequency of the domain repeats, or by the combinations of unique domains they contained, and subsequently compared across species.

### Gene expression analysis of CAZyme-coding genes in *E. grandis* and *P. trichocarpa*

In previous studies, next generation deep mRNA-sequencing using the Illumina platform was used to quantify the genome-wide expression in the transcriptomes of multiple tissues in *E. grandis* and *P. trichocarpa* [37,48,63,64]. Genome-wide transcriptome data for six tissues in *E. grandis* from Dr. C. Hefer was obtained for analysis of the transcript abundance of all expressed CAZyme genes [63]. The tissues analyzed in this study were: Young leaves, mature leaves, immature xylem, phloem, shoot tips, and flowers of *E. grandis* [48,63]. mRNA-sequencing data from young leaf and immature xylem tissue of *P. trichocarpa* was used for comparison to the *E. grandis* transcriptome [64]*.* The expression levels of every gene in each tissue/organ were averaged across three biological replicates, and filtered for genes containing CAZyme domains in *E. grandis* and *P. trichocarpa* from the dbCAN database [34] for further analysis.

The transcript abundance of genes from mRNA-Seq can be quantified as Fragments Per Kilobase of exon per Million fragments mapped (FPKM) [65]. FPKM parameters K and M are optimized to individual experiments in the software used to assemble the transcriptomes, in this case Cufflinks [66], was used (for more detail, refer to [64]). To infer the investment of expression of CAZyme domain families in each tissue, the total transcript abundance for all genes in each CAZyme domain family was summed, and compared that total to the FPKM expression investment values for the other tissues, using Excel for numerical comparisons and visualization. When calculating total expression investment of domain families, genes annotated with multiple CAZyme domain families were treated differently: If the gene was annotated as consisting solely of repeats of the same CAZyme domain, the total transcript abundance of the entire gene was added once to the CAZyme domain family total transcript abundance. Therefore repeat domains of the same CAZyme family were ignored when calculating CAZyme domain family specific transcript abundance. If the gene was annotated as having multiple domains from different CAZyme domain families, the transcript abundance of that gene was added separately to each domain family once. For example, a gene annotated as having domains “X-X-Y”, would have the FPKM value of the gene added once to “family X expression investment total”, and once to “family Y expression investment total”.

## Additional files

Additional file 1: Table S1. Relative standard deviation (RSD) (absolute co-efficient of variation) between plant species. Additional file 2:
**Excel file: CAZyme domain family frequency across twenty-two plant species.**
Additional file 3: Figure S1. Domain family frequency distribution across twenty-two species. Additional file 4: Figure S2. Number of CAZy domains in complex CAZy domain containing proteins across ten representative plant species. Additional file 5:
**Excel file: CAZyme domain containing protein complexity summary in 10 plant species.**
Additional file 6: Figure S3. Venn diagram of CAZyme domain unique combinations within complex proteins in five eudicots. Additional file 7:
**Excel file: Frequency of unique CAZyme domain combinations in complex proteins in 10 plant species (in separate tabs).**
Additional file 8:
**Excel file: Expressed CAZyme domain containing proteins (FPKM) and domain content in** ***E. grandis.***
Additional file 9:
**Excel file: CAZyme domain family expression in FPKM with standard deviation in** ***E. grandis.***
Additional file 10: Figure S4. GH domain family expression levels across six tissues in *E. grandis* in FPKM. Additional file 11: Figure S5. PL domain family expression levels across six tissues in E. grandis in FPKM. Additional file 12: Figure S6. CE domain family expression level across six tissues in E. grandis in FPKM. Additional file 13: Figure S7. CBM domain family expression level across six tissues in *E. grandis* in FPKM. Additional file 14: Figure S8. Comparative expression patterns of GH domain families in *E. grandis* and *P. trichocarpa*. Additional file 15: Figure S9. Comparative expression patterns of PL domain families in *E. grandis* and *P. trichocarpa.*
Additional file 16: Figure S10. Comparative expression patterns of CE domain families in *E. grandis* and *P. trichocarpa.*
Additional file 17: Figure S11. Comparative expression patterns of CBM domain families in *E. grandis* and *P. trichocarpa.*
Additional file 18:
**Python script domain_counter.py: Used to count the frequency of multiple domains in all species for all families across columns. **Comments included in file. Additional file 19:
**Python script domain_pull.py: Used to sort gene frequency based on domain family. **Comments included in file.

## Abbreviations

CAZymes: Carbohydrate Active enZymes
GT: Glycosyl transferase
GH: Glycosyl hydrolase
CE: Carbohydrate esterase
PL: Polysaccharide lyase
CBM: Carbohydrate binding module
CW: Cell wall
SCW: Secondary cell wall
FPKM: Fragments Per Kilobase of exon per Million fragments mapped
XTH: Xyloglucan endotransglycosylase/transferase
CESA: Cellulose synthase A
