# Supplementary material for: Comparative analysis of plant carbohydrate active enZymes and their role in xylogenesis
Source: BMC Genomics. 2015 May 22;16(1):402. doi: 10.1186/s12864-015-1571-8 (PMC4440533; doi:10.1186/s12864-015-1571-8)
Supplement: Additional file 1: Table S1. — Relative standard deviation (RSD) (absolute co-efficient of variation) between plant species. [file 12864_2015_1571_MOESM1_ESM.pdf]

Table S1 Relative standard deviation (RSD) (absolute co-efficient of variation) between plant species.

	GH RSD%	GT RSD%	PL RSD%	CE RSD%	CBM RSD%
**Group 1: Green algae**	4.88207335	5.28067996	9.7954098	3.25097246	15.1713114
**Group 2: Lycophytes and bryophytes**	2.05345889	0.90480126	27.1660388	10.278046	2.64240608
**Group 3: Monocots**	8.70570272	6.78586237	37.6825923	6.5667243	13.8290614
**Group 4: Eudicots**	5.44328404	3.75275016	11.8457336	4.9094062	8.40329611
