# Supplementary figures and images for: Comparative analysis of plant carbohydrate active enZymes and their role in xylogenesis
Source: BMC Genomics. 2015 May 22;16(1):402. doi: 10.1186/s12864-015-1571-8 (PMC4440533; doi:10.1186/s12864-015-1571-8)

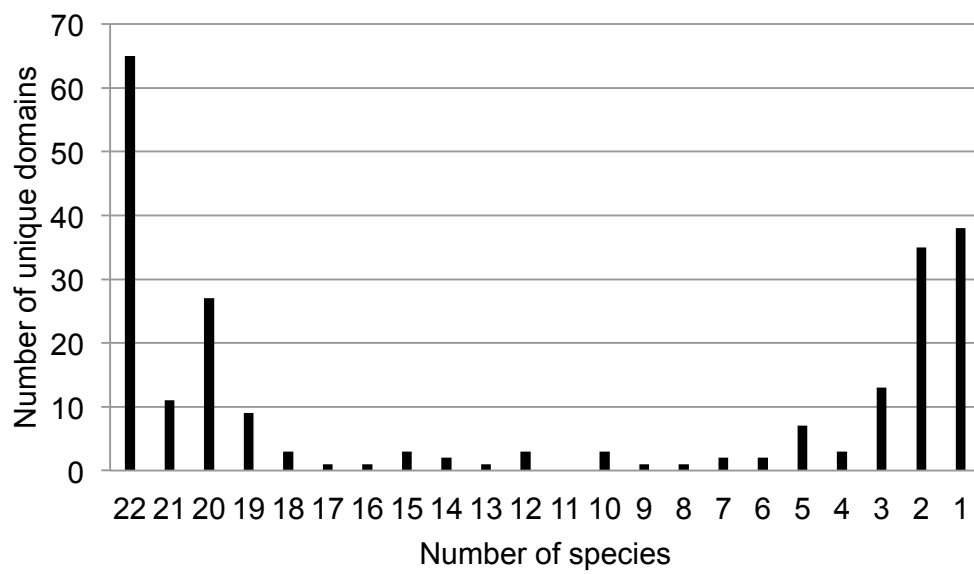

**Figure S1** Domain family frequency distribution across twenty-two species

Supplement: Additional file 3: Figure S1. — Domain family frequency distribution across twenty-two species. [file 12864_2015_1571_MOESM3_ESM.pdf]

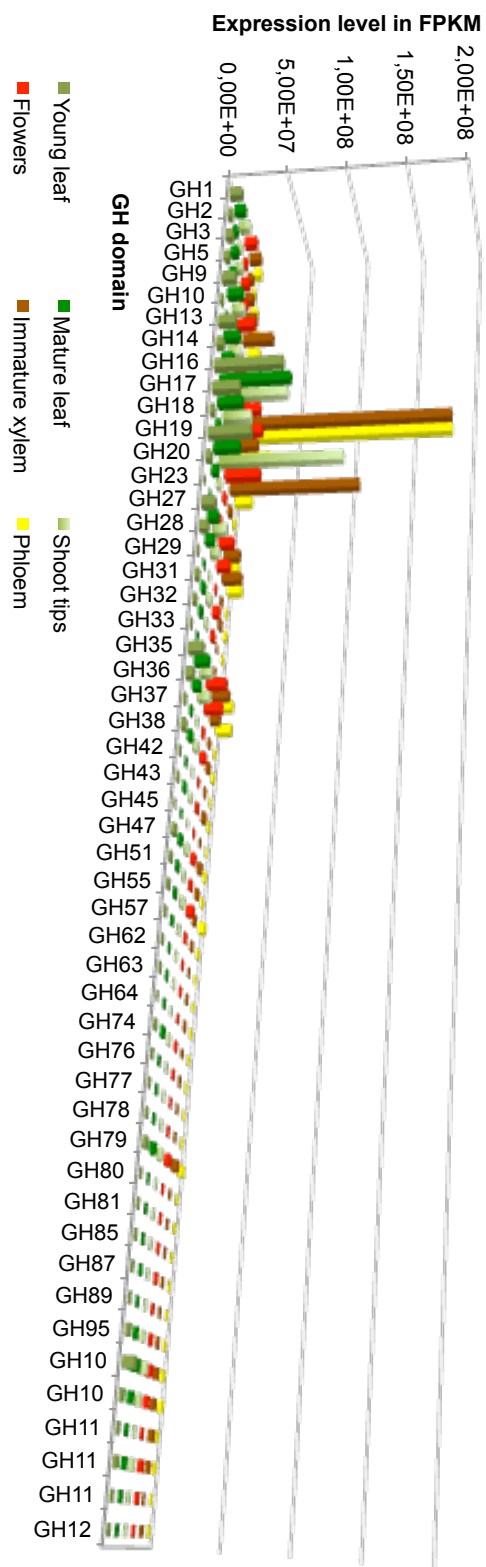

**Figure S4** GH domain family expression levels across six tissues in *E. grandis* in FPKM.

Supplement: Additional file 10: Figure S4. — GH domain family expression levels across six tissues in E. grandis in FPKM. [file 12864_2015_1571_MOESM10_ESM.pdf]

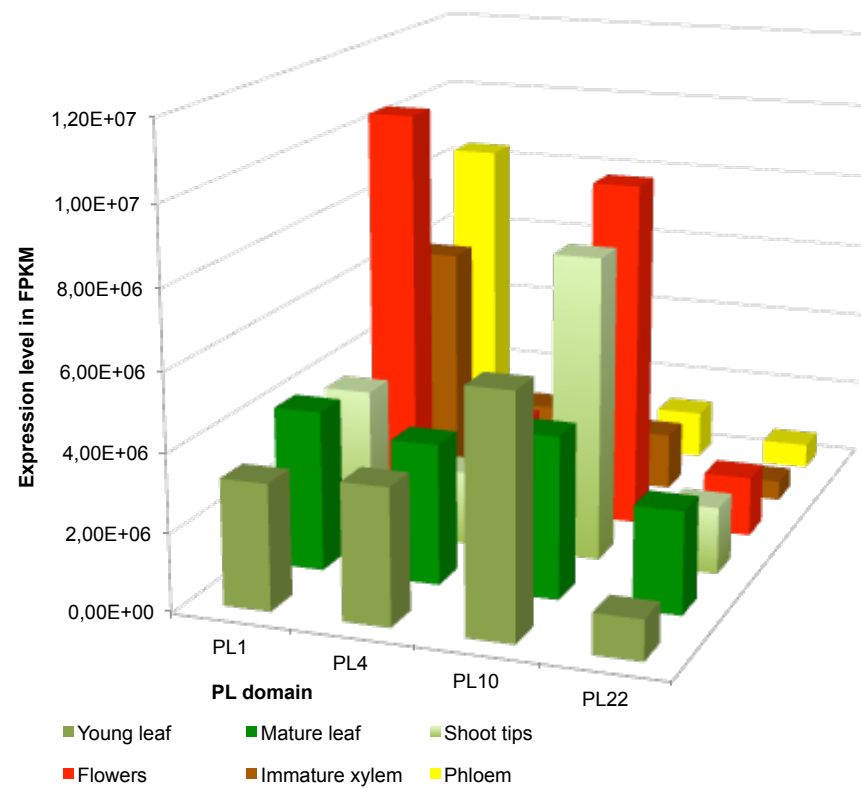

**Figure S5** PL domain family expression levels across six tissues in *E. grandis* in FPKM.

Supplement: Additional file 11: Figure S5. — PL domain family expression levels across six tissues in E. grandis in FPKM. [file 12864_2015_1571_MOESM11_ESM.pdf]

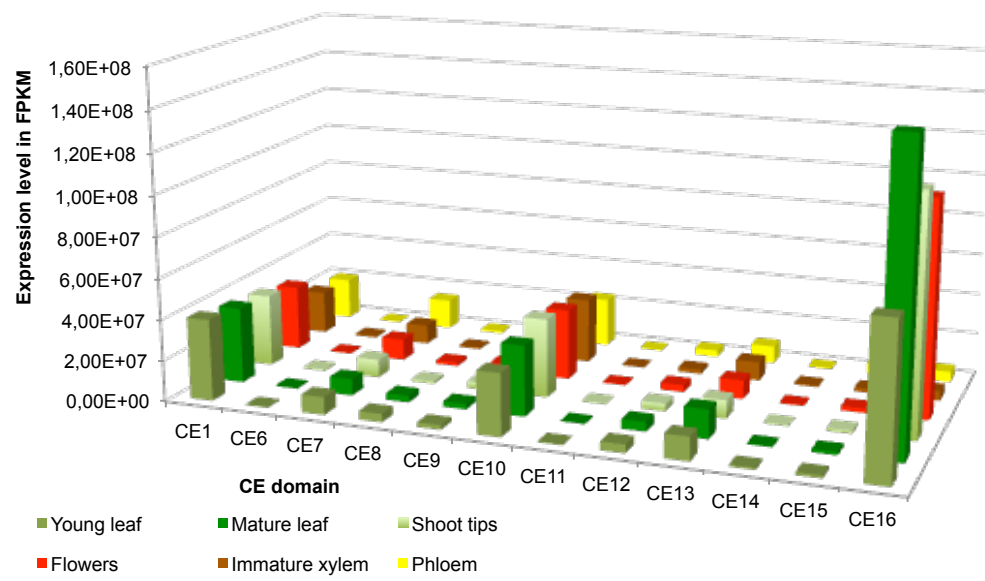

**Figure S6** CE domain family expression level across six tissues in *E. grandis* in FPKM.

Supplement: Additional file 12: Figure S6. — CE domain family expression level across six tissues in E. grandis in FPKM. [file 12864_2015_1571_MOESM12_ESM.pdf]

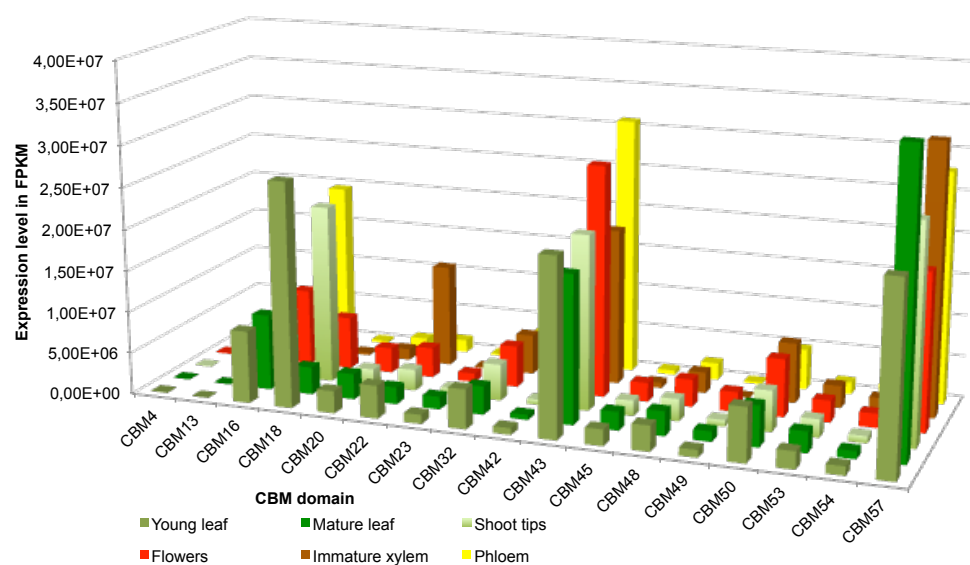

**Figure S7** CBM domain family expression level across six tissues in *E. grandis* in FPKM.

Supplement: Additional file 13: Figure S7. — CBM domain family expression level across six tissues in E. grandis in FPKM. [file 12864_2015_1571_MOESM13_ESM.pdf]

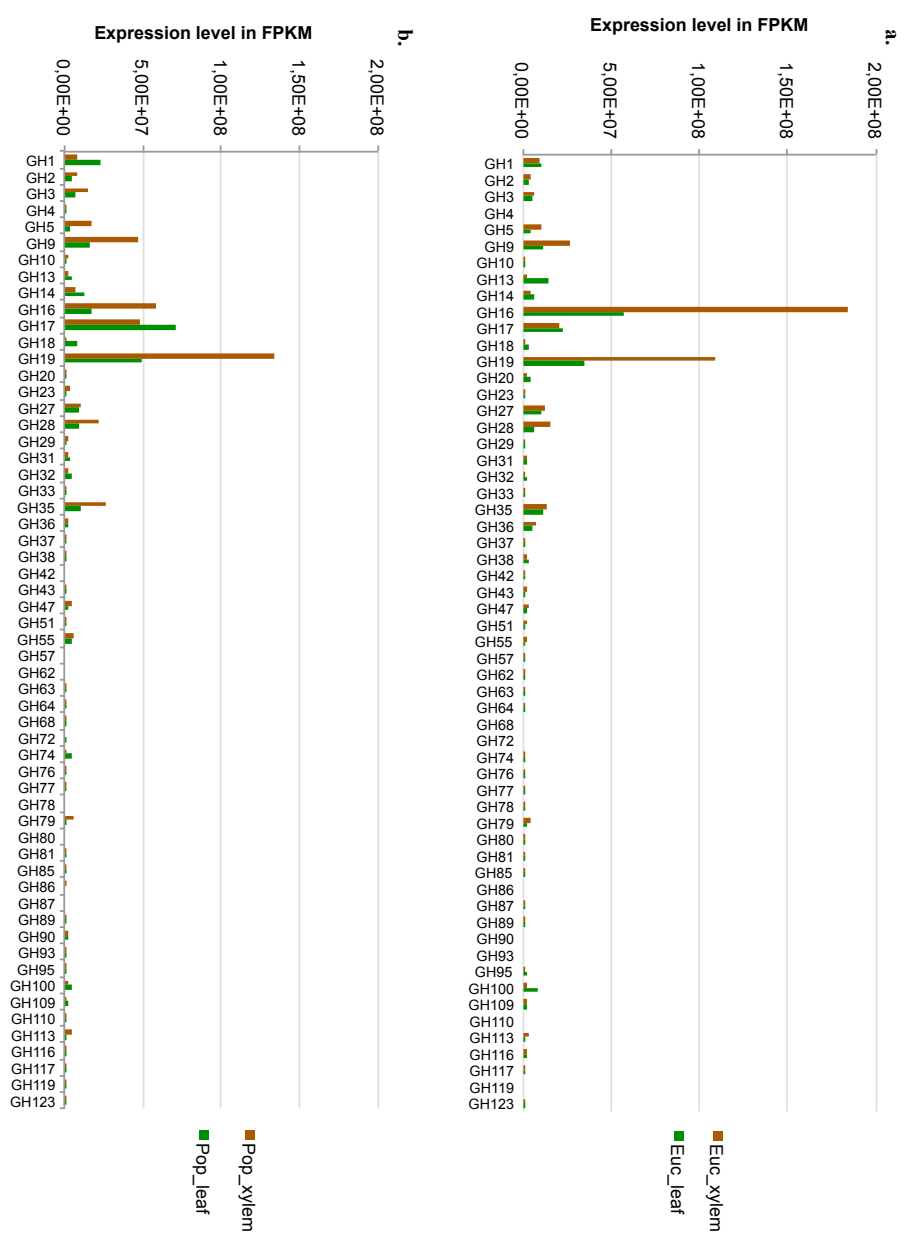

**Figure S8** Comparative expression patterns of GH domain families in *E. grandis* and *P. trichocarpa*.

Supplement: Additional file 14: Figure S8. — Comparative expression patterns of GH domain families in E. grandis and P. trichocarpa. [file 12864_2015_1571_MOESM14_ESM.pdf]

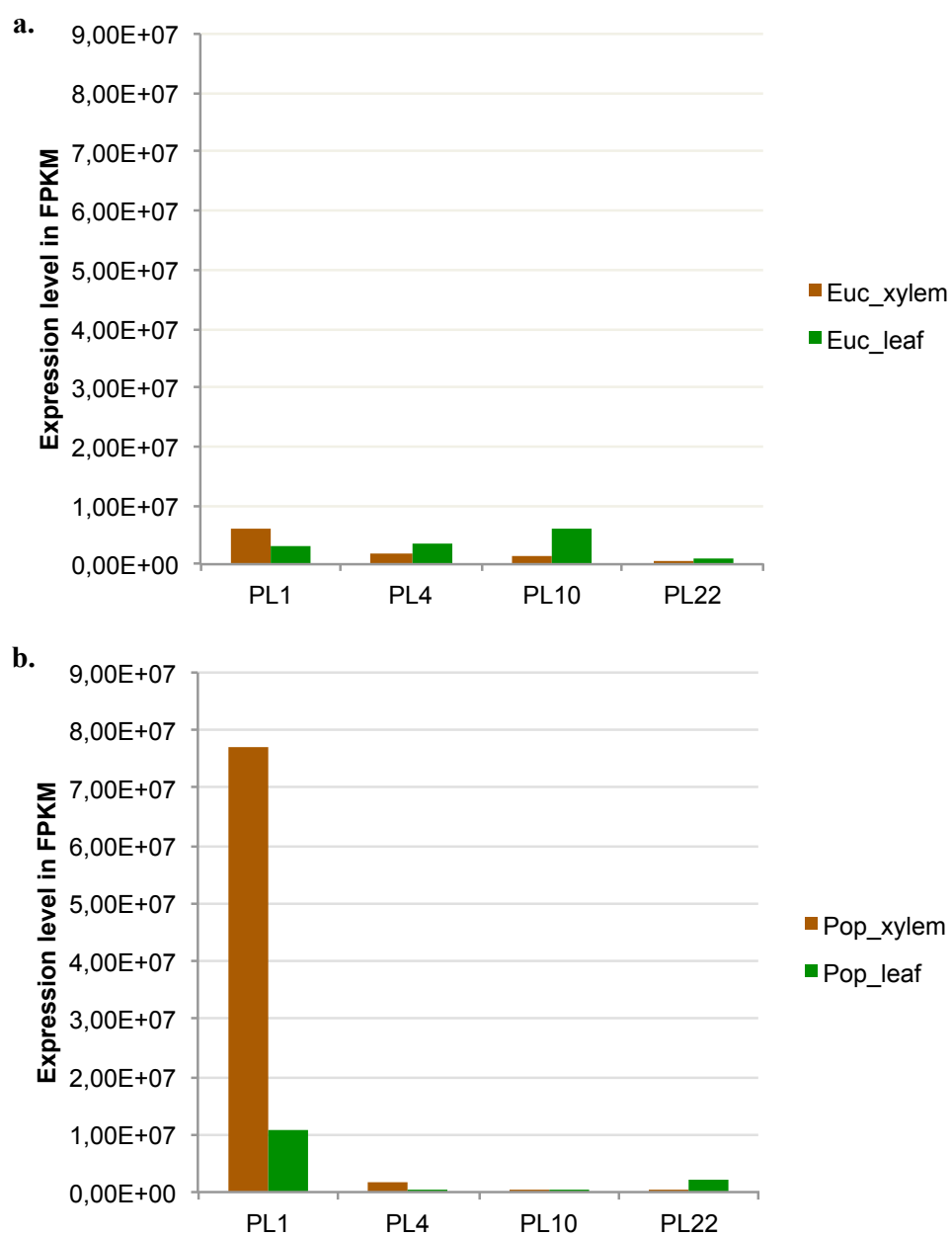

**Figure S9** Comparative expression patterns of PL domain families in *E. grandis* and *P. trichocarpa*.

Supplement: Additional file 15: Figure S9. — Comparative expression patterns of PL domain families in E. grandis and P. trichocarpa. [file 12864_2015_1571_MOESM15_ESM.pdf]

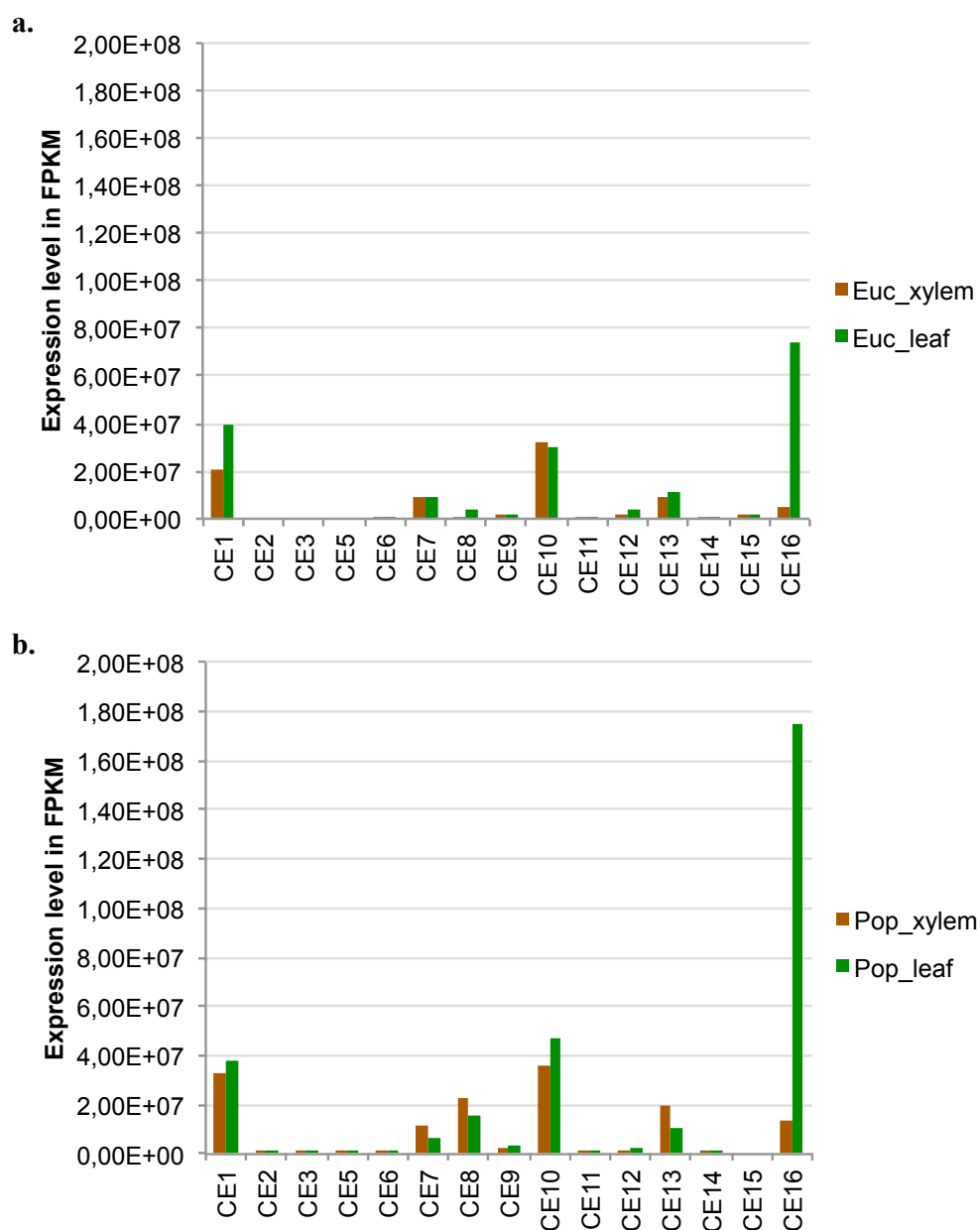

**Figure S10** Comparative expression patterns of CE domain families in *E. grandis* and *P. trichocarpa*.

Supplement: Additional file 16: Figure S10. — Comparative expression patterns of CE domain families in E. grandis and P. trichocarpa. [file 12864_2015_1571_MOESM16_ESM.pdf]
