# Supplementary material for: Comparative analysis of plant carbohydrate active enZymes and their role in xylogenesis
Source: BMC Genomics. 2015 May 22;16(1):402. doi: 10.1186/s12864-015-1571-8 (PMC4440533; doi:10.1186/s12864-015-1571-8)
Supplement: Additional file 4: Figure S2. — Number of CAZy domains in complex CAZy domain containing proteins across ten representative plant species. [file 12864_2015_1571_MOESM4_ESM.pdf]

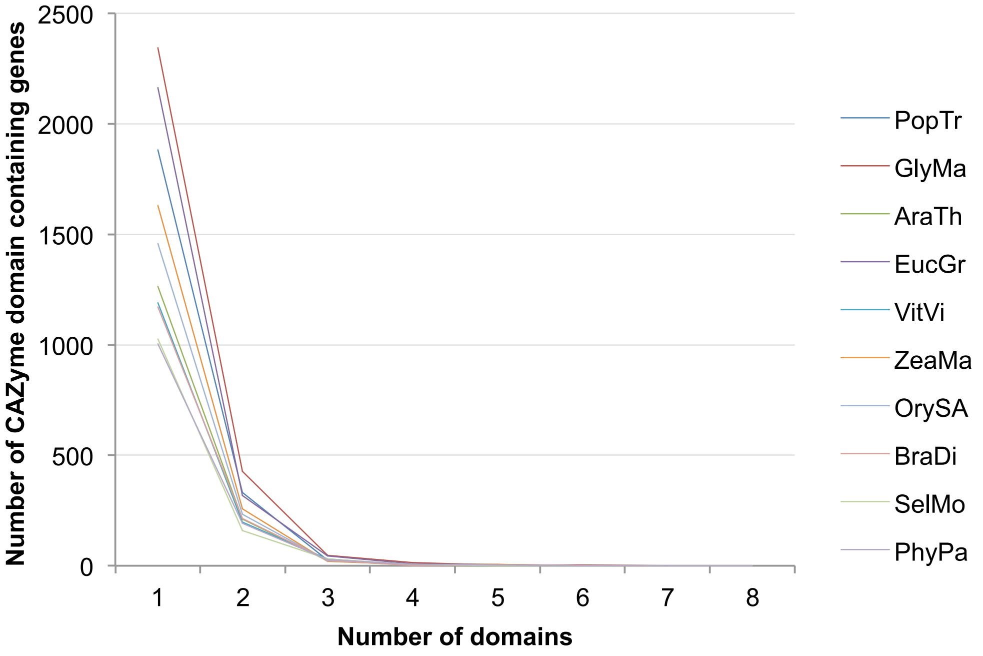

Figure S2 Number of CAZy domains in complex CAZy domain containing proteins across ten representative plant species.
