# Supplementary material for: Comparative analysis of plant carbohydrate active enZymes and their role in xylogenesis
Source: BMC Genomics. 2015 May 22;16(1):402. doi: 10.1186/s12864-015-1571-8 (PMC4440533; doi:10.1186/s12864-015-1571-8)
Supplement: Additional file 6: Figure S3. — Venn diagram of CAZyme domain unique combinations within complex proteins in five eudicots. [file 12864_2015_1571_MOESM6_ESM.pdf]

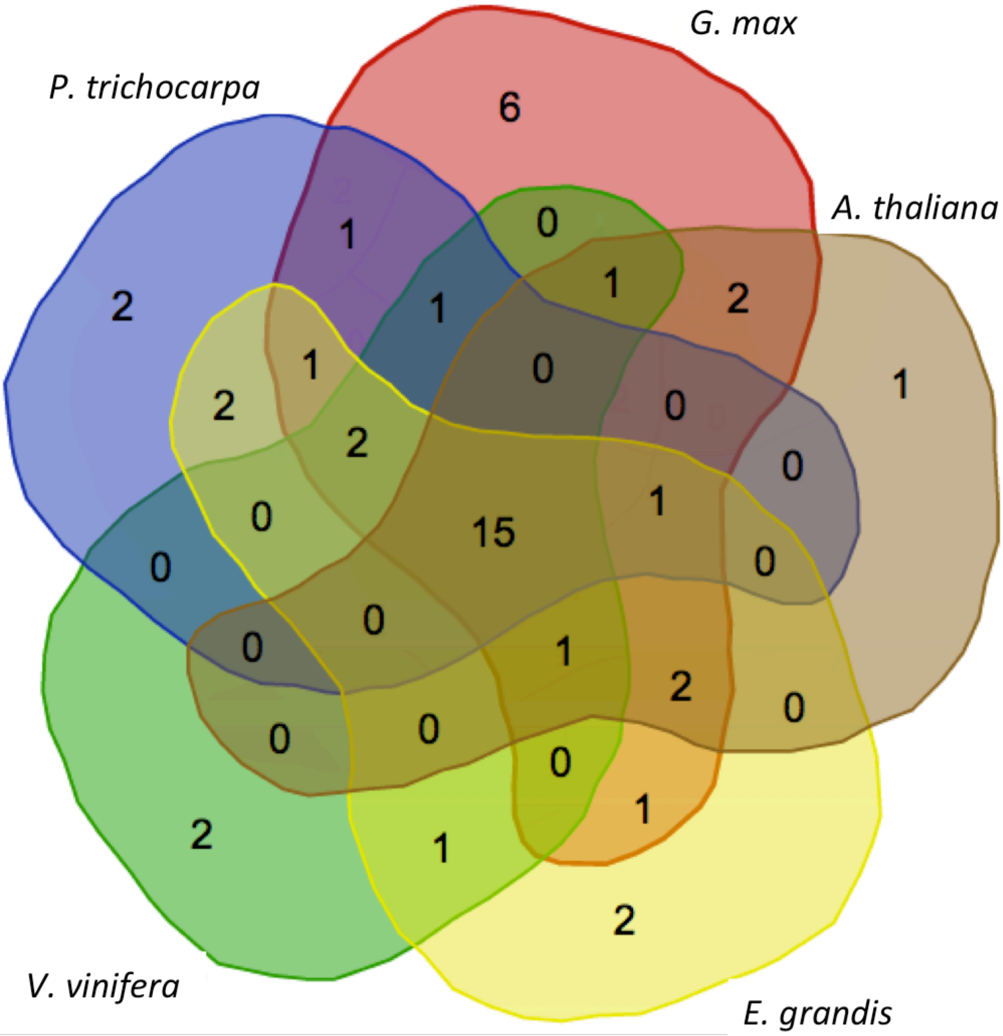

Figure S3 Venn diagram of CAZyme domain unique combinations within complex proteins in five eudicots.
