# Supplementary material for: Comparative analysis of plant carbohydrate active enZymes and their role in xylogenesis
Source: BMC Genomics. 2015 May 22;16(1):402. doi: 10.1186/s12864-015-1571-8 (PMC4440533; doi:10.1186/s12864-015-1571-8)
Supplement: Additional file 17: Figure S11. — Comparative expression patterns of CBM domain families in E. grandis and P. trichocarpa. [file 12864_2015_1571_MOESM17_ESM.pdf]

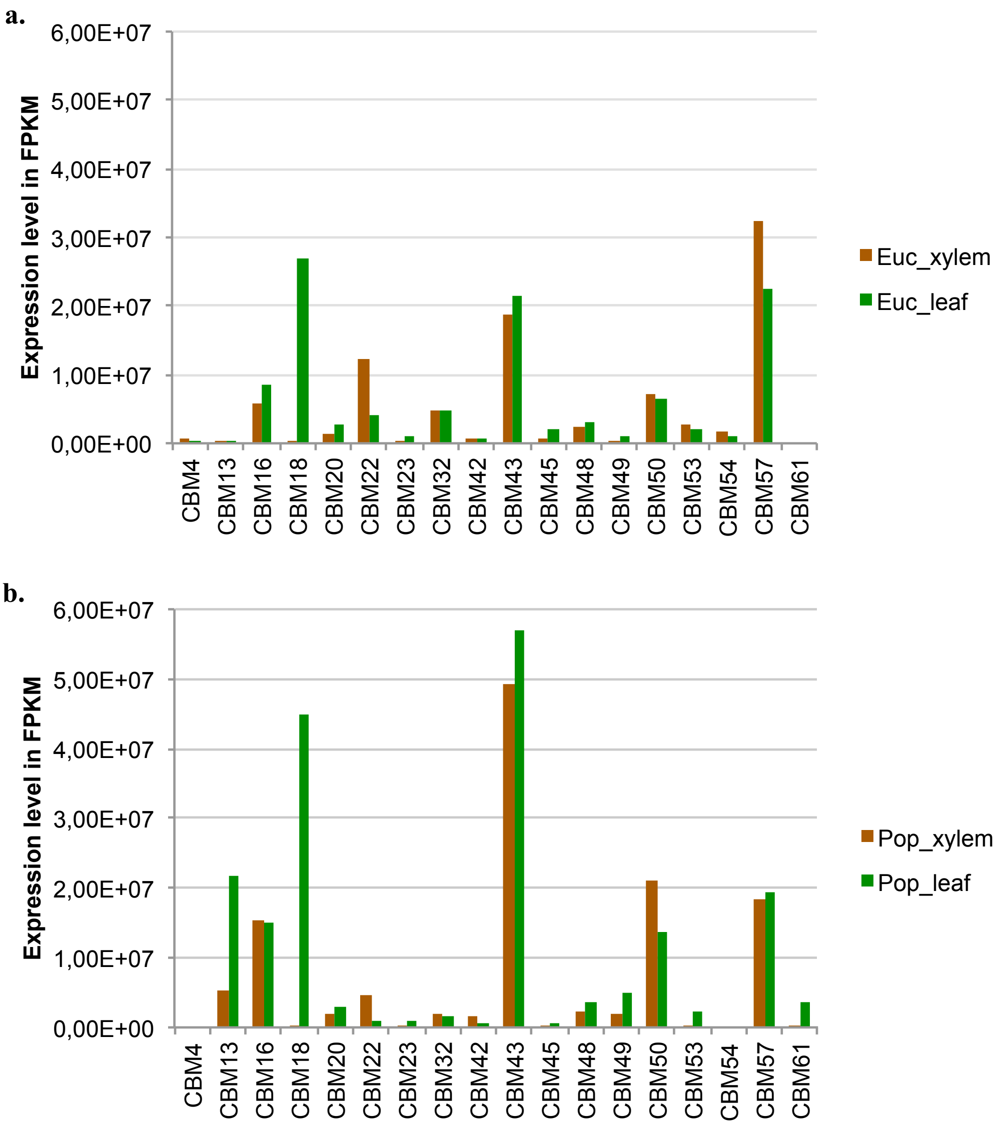

Figure S11 Comparative expression patterns of CBM domain families in *E. grandis* and *P. trichocarpa*.
